# A Suitable Streptomycin-Resistant Mutant for Constructing Unmarked In-Frame Gene Deletions Using *rpsL* as a Counter-Selection Marker

**DOI:** 10.1371/journal.pone.0109258

**Published:** 2014-09-30

**Authors:** Yu-Kuo Tsai, Ci-Hong Liou, Jung-Chung Lin, Ling Ma, Chang-Phone Fung, Feng-Yee Chang, L. Kristopher Siu

**Affiliations:** 1 Institute of Infectious Diseases and Vaccinology, National Health Research Institutes, Miaoli, Taiwan; 2 Division of Infectious Diseases and Tropical Medicine, Department of Internal Medicine, Tri-Service General Hospital, National Defense Medical Center, Taipei, Taiwan; 3 Section of Infectious Diseases, Department of Medicine, Taipei Veterans General Hospital and National Yang-Ming University, Taipei, Taiwan; 4 Taiwan Centres for Disease Control, Taipei, Taiwan; 5 Graduate Institute of Basic Medical Science, China Medical University, Taichung, Taiwan; Baylor College of Medicine, United States of America

## Abstract

The streptomycin counter-selection system is a useful tool for constructing unmarked in-frame gene deletions, which is a fundamental approach to study bacteria and their pathogenicity at the molecular level. A prerequisite for this system is acquiring a streptomycin-resistant strain due to *rpsL* mutations, which encodes the ribosomal protein S12. However, in this study no streptomycin resistance was found to be caused by *rpsL* mutations in all 127 clinical strains of *Klebsiella pneumoniae* isolated from liver abscess patients. By screening 107 spontaneous mutants of streptomycin resistance from a clinical strain of *K. pneumoniae*, nucleotide substitution or insertion located within the *rpsL* was detected in each of these strains. Thirteen different mutants with varied S12 proteins were obtained, including nine streptomycin-dependent mutants. The virulence of all four streptomycin-resistant mutants was further evaluated. Compared with the parental strain, the K42N, K42T and K87R mutants showed a reduction in growth rate, and the K42N and K42T mutants became susceptible to normal human serum. In the mice LD_50_ (the bacterial dose that caused 50% death) assay, the K42N and K42T mutants were ∼1,000-fold less lethal (∼2×10^5^ CFU) and the K87R mutant was ∼50-fold less lethal (∼1×10^4^ CFU) than the parental strain (∼2×10^2^ CFU). A K42R mutant showed non-observable effects on the above assays, while this mutant exhibited a small cost (*P*<0.01) in an *in vitro* growth competition experiment. In summary, most of the *K. pneumoniae* strains with streptomycin resistance caused by *rpsL* mutations are less virulent than their parental strain in the absence of streptomycin. The K42R mutant showed similar pathogenicity to its parental strain and should be one of the best choices when using *rpsL* as a counter-selection marker.

## Introduction

Streptomycin, the first aminoglycoside antibiotic, was reported in 1944 [1] and can inhibit protein synthesis by binding to the 30S ribosomal subunit. The most common mechanisms contributing to streptomycin resistance are aminoglycoside-modifying enzymes and target gene mutations [2], while 16S rRNA methylases, which have recently emerged, confer high-level resistance to all clinically available aminoglycosides except streptomycin [3]–[5]. Adenylyltransferase encoded by *aadA* and phosphoryltransferase encoded by *strA* or *strB* have been demonstrated to be responsible for streptomycin resistance and are widely disseminated among *Enterobacteriaceae* [2], [6], [7]. On the other hand, streptomycin resistance caused by its target genes is mediated by mutations in 16S rRNA encoded by *rrs* or ribosomal protein S12 encoded by *rpsL* [8]. However, a single mutation in an *rrs* gene has only a limited effect on bacteria with multicopy *rrs* gene [9], such as that in *Escherichia coli* and *Klebsiella pneumoniae*. The mutations of the S12 protein have often been found to confer a high-level streptomycin resistance by preventing streptomycin binding and/or conferring ribosomal hyperaccuracy [8], [10], [11]. Some mutations could cause a strongly hyperaccurate phenotype and lead to streptomycin dependence [11]. The streptomycin-dependent mutants need streptomycin to keep their survival because their ribosomal proteins require the binding of streptomycin for properly function during protein synthesis [11].

*K. pneumoniae* is a common cause of infections. Most community-acquired *K. pneumoniae* infections cause pneumonia or urinary tract infections, while a distinct invasive syndrome that causes liver abscesses has been increasingly reported in the past two decades [12]. The construction of unmarked in-frame gene deletions in bacteria is a fundamental approach to study bacteria at a molecular level, including the study of their pathogenicity. Counter-selection markers have been used for the positive selection of the deletion mutant, and the streptomycin counter-selection system is one of the most frequently used [13]. A prerequisite for this system is to acquire a streptomycin-resistant strain due to *rpsL* mutations. When both wild-type and mutant alleles of *rpsL* are expressed in the same strain, it confers a streptomycin-sensitive phenotype in streptomycin-resistant strains, which enables positive selection with streptomycin to detect the loss of the vector that contains the wild-type allele of *rpsL* [13], [14]. Previous studies have demonstrated that *rpsL* can be used as a counter-selection marker in various bacteria, such as *K. pneumoniae* [15]–[20], *Vibrio cholera* [14], *Borrelia burgdorferi* [21], *Corynebacterium glutamicum* [22], *Streptococcus pneumoniae* [23] or mycobacteria [24], [25], but little is known about which streptomycin resistance-inducing *rpsL* mutations should be used. Previous studies have found that some *rpsL* mutations could reduce the virulence of bacteria [26], [27], and an *rpsL* mutant of *K. pneumoniae* showing lower virulence than its parental liver abscess isolate has been selected for use with this technology [17]–[20]. However, our results demonstrated that *rpsL* mutations were rare in *K. pneumoniae* that was isolated from liver abscess patients, and previous studies have showed that different protein expression levels could be caused by *rpsL* mutations [26], [28]. These results suggest that a low or no-cost *rpsL* mutant should be chosen for the use of *rpsL* as a counter-selection marker. In this study, thirteen different streptomycin resistance-inducing mutations of the S12 protein were obtained, and the costs of these mutations were evaluated.

## Materials and Methods

### Ethics Statement

All studies with human blood samples were conducted within the National Defense Medical Center (Taiwan), and blood samples from healthy volunteers were obtained after written informed consent was provided. *K. pneumoniae* strains isolated from patients with liver abscesses were received from already-existing collections [29], [30]. The use of these samples was approved by the Institutional Review Board of the National Defense Medical Center (Taiwan), and the identification number is B-102-13. All samples were anonymized and identified only by study subject number.

All animal studies were conducted in strict accordance with the recommendations in the Guide for the Care and Use of Laboratory Animals of the National Research Council. The protocol was approved by the Institutional Animal Care and Use Committee of the National Defense Medical Center (Taiwan), and the identification number is IACUC-13-171.

### Strains, Growth Rate, Streptomycin Susceptibility and Resistance Genes

The 127 strains of *K. pneumoniae* investigated were isolated from liver abscess patients from 2002 to 2009 [29], [30], and 48, 55 and 24 isolates were collected from Taiwan, Singapore and Hong Kong, respectively. Unless otherwise noted, *K. pneumoniae* and its derivatives were cultured at 37°C in Luria-Bertani (LB) broth or brain heart infusion (BHI) broth with appropriate antibiotics. The growth rate was evaluated using a Bioscreen C MBR, an automated microbiology growth analysis system (Oy Growth Curves Ab, Helsinki, Finland). Cells from overnight cultures were transferred to fresh LB broth without or with streptomycin (50 or 500 µg/ml) to give an initial OD_600_ of 0.005. The cultures were incubated in the Bioscreen C system at 37°C with continuous shaking between measurements, and the growth was quantified every 20 min based on the OD_600_. Each experimental run was conducted with an associated negative control sample containing blank medium and a positive control sample with wild-type cells. The maximum doubling time was determined from the logarithms of the values measured in the mid-exponential phase. Minimal inhibitory concentrations (MICs) of streptomycin were determined using the E-test (Biodisk AB, Sweden). To evaluate the reasons for streptomycin resistance, *aadA*, *strA*, *strB* and *rpsL* were detected via PCR and then sequenced using primer pairs as shown in Table S1. The specific primers for *aadA*, *strA* and *strB* were designed based on the conserved regions of their open reading frames in previous studies [7], [31], while the specific primers for *rpsL* were designed according to the conserved regions of its franking region in this study.

### Selection and Identification of Spontaneous Mutations

*K. pneumoniae* NVT1001, capsular serotype 1, was one of the isolates from the liver abscess patients in Taiwan. This strain was grown in LB broth at 37°C to the late-exponential growth phase; the culture was then diluted 10^−8^-fold in 4 ml fresh LB broth. After being incubated at 37°C overnight until the stationary growth phase, at which point the viable cell number was estimated to be ∼1.6×10^9^ cells/ml, the cells of 1 ml culture were harvested via centrifugation at 12,000 *g* for 5 min, washed prior to being resuspended in 0.1 ml sterile physiological saline (0.9% sodium chloride), and then spread on LB agar plates supplemented with 50 or 500 µg/ml streptomycin. The cells were incubated at 37°C, and spontaneous mutants were obtained from colonies that arose within 3 days. Independent cultures were grown and plated as described above. Only one mutant colony was picked from each culture every day. The DNA fragment containing the complete *rpsL* gene was amplified via PCR using the primers rpsL.for and rpsL.rev (Table S1) for the selected mutants. The mutations in the *rpsL* gene were identified via sequencing and comparison with the nucleotide sequence of the wild-type strain.

### Construction of Revertants

Plasmid pUT-kmy, which consists of an R6K origin of replication, an mobRP4 origin of transfer, and a kanamycin resistance cassette [32], was ligated with a *sacB* gene to generate plasmid pUT-KB for constructing revertants. Plasmid pUT-KB is a suicide vector containing a counter-selection marker, *sacB*, which originates from *Bacillus subtilis* [13]. When this gene is expressed on the integrated pUT-KB, it confers a sucrose-sensitivity phenotype, which enables positive selection with sucrose to detect the loss of the vector.

The allelic exchange method was used to restore the wild-type *rpsL* gene in the *K. pneumoniae* K42R, K42N, K42T, and K87R mutants (Figure S1). Briefly, DNA fragments of the entire *rpsL* with their flanking regions were amplified from *K. pneumoniae* NVT1001 using PCR with the primers ApaL.for and ApaL.rev (Table S1). The 2.4-kb PCR fragment generated was digested with ApaLI and then cloned into pUT-KB that was similarly digested, resulting in plasmid pRpsL-WT. For homologous recombination, plasmid pRpsL-WT was then transformed into *E. coli* S17-1 λ*pir* [14] using the heat shock method and mobilized into the *K. pneumoniae* K42R, K42N, K42T and K87R mutants via conjugation. Single-crossover strains were selected from brilliant green containing inositol-nitrate-deoxycholate (BIND) plates supplemented with kanamycin (50 µg/ml), while the growth of the non-*K. pneumoniae* strains was effectively suppressed on the BIND plates [33]. The kanamycin-resistant transconjugant was selected, and the insertion of pRpsL-WT was verified via PCR. After being incubated in 20 ml BHI for 6 hours in the absence of kanamycin at 37°C, the fully grown cultures were spread onto LB plates supplemented with 10% sucrose. After double crossover occurred, the sucrose-resistant and kanamycin-sensitive colonies were selected, and the restorations of wild-type *rpsL* gene were confirmed via DNA sequencing.

### *In Vitro* Growth Competition Assay

Competitive growth *in vitro* was performed as previously described [34], [35] with minor modifications. Briefly, cells were grown overnight in LB broth at 37°C to the stationary phase, and equal densities of *K. pneumoniae* NVT1001 and one of its streptomycin-resistant mutants were mixed and then diluted 2^17^-fold into 4 ml of fresh LB broth each to a final concentration of ∼6×10^3^ CFU/ml. This culture was incubated at 37°C with 200 rpm shaking for 24 hours to complete a growth cycle (∼1.6×10^9^ cells/ml, ∼17 generations). Each successive growth cycle was initiated by diluting the mixture 2^17^-fold into 4 ml of fresh LB broth. After the initial mixing and each growth cycle, appropriate dilutions of the mixture were plated on LB agar plates for colony counts. The number of streptomycin-resistant bacteria was determined by plating the mixture on LB agar plates supplemented with 500 µg/ml streptomycin. The number of parental streptomycin-susceptible bacteria was calculated as the total number of bacteria minus the number of streptomycin-resistant bacteria. Each competition assay was performed in triplicates of four competition cycles, and the serial dilutions were plated in triplicate The difference in fitness between two competing strains was calculated using the following function as previously described [34], [35]:
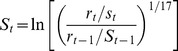
where *r_t_* and *s_t_* denote the number of drug-resistant and drug-susceptible cells at a given time *t*, respectively, and *r_t−1_* and *s_t−1_* denote the number of drug-resistant and drug-susceptible cells at the preceding time point, respectively. The quotient of the ratios of the cell numbers was standardized with the exponent 1/17 because cell numbers were determined approximately every 17 generations. *S_t_* is called the selection coefficient at time *t*. *S_t_* is equal to 0 if there is no difference in fitness between the competing strains. If antibiotic resistance reduces bacterial fitness, *S_t_* is negative, while *S_t_* is positive if resistance increases bacterial fitness. The data are presented as relative bacterial fitness (fit*_t_*), which was defined by Sander *et al.* as fit*_t_* = 1+*S_t_* [34].

### Neutrophil Phagocytosis Assay

Phagocytosis was measured using a standard assay [36]. A FACScan (Becton Dickinson Immunocytometry Systems, San Jose, CA, USA) was used to measure the phagocytic rate. The labeling of bacteria with fluorescein isothiocyanate was performed as described by Heinzelmann *et al.* [36], and the isolation of neutrophils from three healthy volunteers was performed as previously described [37]. A mixture of labeled bacteria, the neutrophil suspension, the pooled normal human serum and PBS was incubated in a shaking water bath at 37°C. The percentage of neutrophils that had phagocytosed bacteria was counted at 15 and 30 min. An unincubated tube served as the 0 min time point. The experimental procedures and FACS settings have been described previously [38].

### Serum Bactericidal Assay

Normal human serum (NHS) pooled from healthy volunteers was divided into equal volumes and stored at −70°C prior to use. The serum bactericidal activity was measured using the method described by Podschun *et al.* [39], which was modified using a 10-fold reaction volume and 2-fold bacteria concentration [15]. Briefly, bacteria were grown in BHI broth until an OD_600_ of 0.35 (∼10^8^ cells/ml) was reached. The cultures were washed and then diluted 25-fold using phosphate buffered saline (PBS). Two hundred fifty microliters of the cell suspension and 750 µl of pooled human serum were placed into 1.5 ml Eppendorf tubes, mixed, and incubated at 37°C. To determine the number of viable bacteria, an aliquot of each bacterial suspension was removed immediately and after 3 hours of incubation. The number of viable bacteria was determined via dilution and plating on LB agar for colony counts. The results were expressed as a percentage of the inoculum, and strains with survival rates >100% in NHS after 3 hours of incubation were considered resistant; those with survival rates <100% were considered susceptible. NHS was decomplemented via heating at 56°C for 30 min. The classical and lectin complement pathways were selectively blocked via chelation with 10 mM ethylene glycol-bis(β-aminoethyl ether)-*N,N,N′N′*-tetraacetic acid (EGTA) plus 10 mM MgCl_2_ (NHSMgEGTA) [40]–[42]. To selectively inhibit the alternative complement pathway, factor B was inactivated via the incubation of NHS at 50°C for 20 min (NHS50°C) [42], [43].

### Mouse Lethality Assay

Pathogen-free, 6- to 8-week old, male BALB/c mice were obtained from the National Laboratory Animal Center (Taiwan) and maintained in the pathogen-free vivarium of the Laboratory Animal Center of National Defense Medical Center (Taiwan). The tested bacteria were cultured overnight at 37°C in BHI broth and were then diluted (1∶100) in fresh BHI broth. The culture was incubated until the mid-exponential growth phase, and the cells were then washed once, resuspended in PBS, and adjusted to the desired concentrations according to OD_600_. The actual concentrations were verified by plating the cells to determine viable counts. Six mice for each group were injected intraperitoneally with 0.1 ml of the cell suspension, and the mice were monitored daily for 14 days to measure survival and the severity of illness.

### Statistical Analysis

A two-tailed *t*-test or a log-rank test (for survival analysis) was used for statistical analysis. A *P* value of <0.05 was considered statistically significant.

## Results

### Streptomycin Susceptibility and Resistance Genes

The 127 *K. pneumoniae* strains obtained from the liver abscess patients in different geographic localities were investigated as described in the Materials and Methods. The minimal inhibitory concentrations (MICs) of streptomycin values ranged from 1 to 64 µg/ml, while the MIC_50_ and MIC_90_ of streptomycin were 3 and 6 µg/ml, respectively. Eighteen *K. pneumoniae* strains (MICs≥6 µg/ml) were used to study the mechanisms of streptomycin resistance (Table 1). The *aadA* gene or *strA*-*strB* genes were detected in the 6 strains that had MICs≥12 µg/ml. No streptomycin resistance was found to be caused by the *rpsL* mutations. *K. pneumoniae* NVT1001, with a 2 µg/ml streptomycin MIC value, was used to select spontaneous mutants with streptomycin resistance (Table 2).

**Table 1:** Streptomycin resistance and target genes in 18 *K. pneumoniae* strains with MIC values ranging from 6 to 64 µg/ml.

Gene	Strain number			
	6^b^	12	16	64
*aadA*	0	2	2	0
*strA*+*strB*	0	0	0	2
None^a^	12	0	0	0
Wild-type *rpsL*	12	2	2	2

a The *aadA*, *strA* and *strB* genes were not detected.

b The MIC (µg/ml) of streptomycin was determined using the E-test.

**Table 2:** Characteristics of *K. pneumoniae* NVT1001 and its derived strains.

Strain	Description^a^	Codon change^b^	Isolation frequency	Streptomycin MIC (µg/ml)	Phenotype^c^	Doubling time (min)^d^		
						Sm 0	Sm 50	Sm 500
NVT1001	Clinical isolate (WT)			2	Sm^S^	18.8±0.4	ND	ND
K42R mutant	*rpsL* K42R	128A→G	8/107	>1,024	Sm^R^	19.1±0.4	19.6±0.3	20.2±0.4
K42N mutant	*rpsL* K42N	129G→T	9/107	>1,024	Sm^R^	**24.7±1.1**	24.4±0.7	23.8±0.9
K42T mutant	*rpsL* K42T	128A→C	5/107	>1,024	Sm^R^	**21.7±0.5**	22.1±0.6	22.2±0.4
K87R mutant	*rpsL* K87R	263A→G	3/107	>1,024	Sm^R^	**20.4±0.6**	21.5±0.9	26.4±1.1
P41L mutant	*rpsL* P41L	125C→T	1/107	>1,024; 96^e^	Sm^D^	ND	ND	61.6±3.1
K42Q mutant	*rpsL* K42Q	127A→C	3/107	>1,024; 96^e^	Sm^D^	ND	ND	58.0±2.9
K43E mutant	*rpsL* K43E	130A→G	2/107	>1,024; 96^e^	Sm^D^	ND	ND	68.7±3.5
+K87 mutant	*rpsL* +K87	262-264::AAA	1/107	>1,024; 96^e^	Sm^D^	ND	ND	61.1±2.1
D88E.1 mutant	*rpsL* D88E	267C→A	1/107	>1,024; 96^e^	Sm^D^	ND	ND	61.7±2.6
D88E.2 mutant	*rpsL* D88E	267C→G	5/107	>1,024; 96^e^	Sm^D^	ND	ND	61.5±2.9
P90Q mutant	*rpsL* P90Q	272C→A	2/107	>1,024; 8^e^	Sm^D^	ND	38.2±1.2	28.9±1.2
P90R mutant	*rpsL* P90R	272C→G	1/107	>1,024; 6^e^	Sm^D^	ND	45.5±2.4	36.1±2.5
P90L mutant	*rpsL* P90L	272C→T	36/107	>1,024; 8^e^	Sm^D^	ND	50.2±3.6	53.3±2.5
G91D mutant	*rpsL* G91D	275G→A	30/107	>1,024; 8^e^	Sm^D^	ND	47.7±1.5	53.0±1.9
K42R revertant	WT-*rpsL* replacement strain of K42R mutant			2	Sm^S^	18.9±0.5	ND	ND
K42N revertant	WT-*rpsL* replacement strain of K42N mutant			2	Sm^S^	19.1±0.8	ND	ND
K42T revertant	WT-*rpsL* replacement strain of K42T mutant			2	Sm^S^	18.6±0.5	ND	ND
K87R revertant	WT-*rpsL* replacement strain of K87R mutant			2	Sm^S^	19.0±0.6	ND	ND

a WT, wild type; Amino acid replacements are listed; +, an insertion.

b Numbering begins with the start codon (ATG) of the ORF.

c Sm^S^, streptomycin-sensitive; Sm^R^, streptomycin-resistant; Sm^D^, streptomycin-dependent.

d Doubling times were measured in Luria-Bertani broth without or with streptomycin (50 or 500 µg/ml) and are the average of three independent experiments. The doubling times of growth in the absence of streptomycin were further bolded when a significant (*P*<0.05) difference was found between NVT1001 and its derived strains. ND, not determined. These strains would not grow in either the presence or absence of streptomycin according to the results of the E-test.

e The minimal streptomycin concentration that enables growth was determined using the E-test, which was also used to determine the streptomycin MICs.

### Isolation and Characteristics of the S12 Mutants

Spontaneous mutants of *K. pneumoniae* that were resistant to streptomycin were selected on LB agar plates supplemented with 50 or 500 µg/ml streptomycin, and 107 mutants were isolated. The *rpsL* genes of all of these mutants were sequenced, and fourteen different mutations on the *rpsL* were obtained, which cause thirteen varied S12 protein (Table 2). The examination of streptomycin resistance for these different mutants using the E-test led to the identification of ten streptomycin-dependent mutants and four streptomycin-resistant mutants (Table 2). Different concentrations (6–96 µg/ml) of streptomycin were needed for the survival of the ten streptomycin-dependent mutants (Table 2).

### Growth Rate and *In Vitro* Growth Competition Assay

Compared to the parental strain cultured in antibiotic-free LB broth, the ten streptomycin-dependent mutants showed ∼1.5- to 3.7-fold slower growth rates (*P*<0.01) when cultured in LB broth supplemented with 500 µg/ml streptomycin. Four of these ten mutants were also cultured in LB broth supplemented with 50 µg/ml streptomycin and the four streptomycin-dependent mutants showed ∼2.0- to 2.7-fold slower growth rates (*P*<0.01) compared to the parental strain cultured in antibiotic-free LB broth. When comparing the four streptomycin-resistant mutants to the parental strain, which were all cultured in antibiotic-free LB broth, the K42R mutant showed a similar growth rate, while the K42N, K42T and K87R mutants showed ∼1.1- to 1.3-fold slower growth rates (*P*<0.05). Unlike other streptomycin-resistant mutants, the K87R mutant showed a ∼1.3-fold slower growth rate (*P*<0.05) when cultured in the presence of 500 µg/ml streptomycin than that without streptomycin. The growth phenotype of the streptomycin-resistant mutants can be restored by replacing the wild-type *rpsL* to these mutants using an allelic exchange method (Table 2 and Figure S1).

A highly sensitive *in vitro* growth competition experiment was used to further evaluate the fitness cost of the four streptomycin-resistant mutants (Table 3). Compared with the parental strain (with a fitness of 1), the K42N, K42T and K87R mutations reduced fitness to ∼0.82, ∼0.85 and ∼0.94 respectively (*P*<0.0001), while the K42R mutation also slightly reduced fitness to ∼0.97 (*P*<0.01). Competition assays were also performed for the four streptomycin-resistant mutants using their revertants as references, and the results demonstrated that the decreased fitness could be restored to original levels following complementation (Table 3).

**Table 3:** Determination of relative fitness via *in vitro* growth competition assay.

Strain	Fitness (fit*_t_* ± SD)^a^	*P* value^b^	N^c^
Relative to NVT1001			
K42R mutant	0.97±0.027	0.0091	12
K42N mutant	0.82±0.011	<0.0001	12
K42T mutant	0.85±0.015	<0.0001	12
K87R mutant	0.94±0.010	<0.0001	12
Relative to its revertant			
K42R mutant	0.98±0.016	0.0005	12
K42N mutant	0.83±0.010	<0.0001	12
K42T mutant	0.86±0.013	<0.0001	12
K87R mutant	0.95±0.016	<0.0001	12

a Fitness relative to *K. pneumoniae* NVT1001 or its revertant (with a fitness of 1); SD, standard deviation.

b *P* value, statistical significance of difference in fitness relative to *K. pneumoniae* NVT1001 or its revertant.

c N, number of fit*_t_* on which the average fit*_t_* value is based.

### Phagocytosis and Serum Bactericidal Assays

No significant difference was found between strain NVT1001 and its four streptomycin-resistant mutants in the neutrophil phagocytosis assay (Table 4). In the serum bactericidal assay, the K42R and K87R mutants both showed resistance to normal human serum (NHS) as their parental strain, while the K42N and K42T mutants showed susceptibility to NHS and a ∼1.8-fold smaller survival rate (*P*<0.05) in decomplemented 75% NHS compared with the parental strain (Table 4). Although NHS resistance was observed in the K87R mutant, this mutant demonstrated a ∼1.3-fold smaller survival rates (*P*<0.05) in 75% NHS compared with the parental strain. Similar result was observed when comparing the survival rates of these two strains cultured in decomplemented 75% NHS. This result indicates that the smaller survival rate of K87R mutant in 75% NHS was mainly caused by its slower growth. The results of serum bactericidal assay have been further validated by testing the revertant strains (Table 4).

**Table 4:** Effect of mutations on phagocytosis and susceptibility to normal human serum (NHS)^a^.

Strain	Ingested bacterial (%)^b^		Survival rate (%)^c^	
	15 min	30 min	75% NHS	Decomplemented
NVT1001	25.6±4.1	46.9±6.0	199±27	204±17
K42R mutant	20.4±3.7	47.7±9.7	198±12	193±16
K42N mutant	25.0±3.9	43.9±7.1	**14±7**	**112±4**
K42T mutant	20.6±5.9	44.3±9.0	**17±8**	**118±9**
K87R mutant	28.2±5.0	45.3±10.9	**151±13**	**142±11**
K42R revertant	ND	ND	194±16	183±11
K42N revertant	ND	ND	203±7	198±13
K42T revertant	ND	ND	201±20	183±5
K87R revertant	ND	ND	206±29	203±10

a Each value represents the means of three independent experiments ± the standard deviation. Boldface numbers indicate a significant (*P*<0.05) difference between NVT1001 and its derived strains. ND, not determined.

b *K. pneumoniae* strains were incubated with neutrophils for 15 or 30 min.

c Percent survival of the cells after 3 h of serum contact. Decomplemented, NHS was decomplemented via heating at 56°C for 30 min.

The two serum-susceptible strains, the K42N and K42T mutants, were used to further investigate the contribution of each complement pathway to complement-mediated bacterial killing. In comparison with culturing in decomplemented 75% NHS, the two mutants cultured in 75% NHS50°C showed a ∼1.2-fold smaller survival rate (*P*<0.05), while their survival rates were strongly reduced (*P*<0.01) in 75% NHSMgEGTA (as in 75% NHS) (Table 5). These results suggest that the alternative pathway has a crucial role in complement activation.

**Table 5:** Comparison of the bactericidal activity of normal human serum (NHS) with different treatments against serum-susceptible *K. pneumoniae* mutants.

NHS^a^	Survival rate (%)^b^	
	K42N mutant	K42T mutant
75% NHS	13±3	17±9
75% NHSMgEGTA	**32±6**	**43±10**
75% NHS50°C	**95±10**	**98±14**
Decomplemented 75% NHS	**120±9**	**115±10**

a NHSMgEGTA, NHS plus inhibition of the classical and lectin complement pathways; NHS50°C, NHS with inhibition of the alternative complement pathway. NHS was decomplemented via heating at 56°C for 30 min.

b Percent survival of cells after 3 h of serum contact. Each value represents the means of three independent experiments ± the standard deviation. Boldface numbers indicate a significant (*P*<0.05) difference in survival rate compared with the same strain incubated in 75% NHS.

### Virulence in Mice

**Figure 1:**
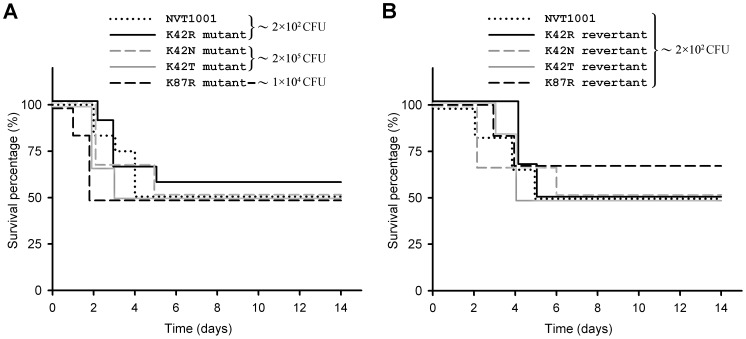
The effect of *rpsL* mutations on mouse lethality. The survival after intraperitoneal injection with 2×10^2^ to 2×10^5^ CFU of *K. pneumoniae* NVT1001, streptomycin-resistant mutant (A) or the revertant (B) was documented over 14 days. The data points represent the percentage of mice surviving in each experimental group over time (n = 6 mice per strain), while the data illustrated for *K. pneumoniae* NVT1001 and the K42R mutant in Figure 1A are pooled from two independent experiments. The LD_50_ of *K. pneumoniae* NVT1001 and the K42R mutant were similar, both approximately 2×10^2^ CFU. The K42N, K42T and K87R mutants were all less virulent than NVT1001, while the LD_50_ of the K42N and K42T mutants were approximately 2×10^5^ CFU and the LD_50_ of the K87R mutant was approximately 1×10^4^ CFU.

To further assess the effects of the *rpsL* mutations on virulence, a mouse peritonitis model was used. A non-significant difference (log-rank test, *P*>0.05) was found between NVT1001 strain and its K42R mutant regarding the survival of 12 mice per strain, and their LD_50_ (the bacterial dose that caused 50% death) were both ∼2×10^2^ CFU (Figure 1A). When the experiment was performed at a higher dose (∼2×10^3^ CFU) in 6 mice per strain, no mice survived within 14 days after being inoculated with the NVT1001 strain or its K42R mutant (data not shown). Compared with the NVT1001-inoculated mice, the mice (n = 6 mice per strain) inoculated with the K42N or K42T mutants showed a ∼1,000-fold increased LD_50_ (∼2×10^5^ CFU), while that inoculated with the K87R mutant showed a ∼50-fold increased LD_50_ (∼1×10^4^ CFU) (Figure 1A). When the experiment was performed at a lower dose in 6 mice per strain, all mice survived within 14 days after being inoculated with K42N (∼2×10^4^ CFU), K42T (∼2×10^4^ CFU) or K87R mutant (∼2×10^2^ CFU) (data not shown). These results have been further confirmed via the intraperitoneal injection of 2×10^2^ CFU of each revertant (K42R, K42N, K42T or K87R revertant) in 6 mice per strain (Figure 1B). A non-significant difference (log-rank test, *P*>0.05) was found between these revertants and NVT1001 strain regarding mouse lethality.

## Discussion

In the 18 *K. pneumoniae* strains that had MICs≥6 µg/ml, streptomycin resistance genes could only be found in the 6 strains that had MICs≥12 µg/ml. This result was similar to the previous study in which the streptomycin epidemiological cut-off values were recommended as WT≤16 µg/ml for *Salmonella* and WT≤8 µg/ml for *E. coli* [2]. The *aadA* gene was found in the four *Klebsiella* strains exhibiting streptomycin MIC 12 or 16 µg/ml. The low-level resistance to streptomycin conferred by *aadA* can also be found in *Salmonella* and *E. coli* [2], [7], [44]. No streptomycin resistance was found to be caused by *rpsL* mutations in any of the 127 clinical strains of *K. pneumoniae*. This result suggests that the mutations of S12 protein were uncommon in *K. pneumoniae* that was isolated from liver abscess patients.

Compared with the parental strain in this study, the K42N and K42T mutants showed reduced virulence, while the K42R mutant showed similar virulence. These results are consistent with those reported in *Salmonella typhimurium* using the competition experiments in mice [27] and those reported in *Erwinia carotovora* using the potato tuber tests [26]. A reduced virulence of the streptomycin-resistant K87R mutant was further found in this study. Compared with the parental strain, the K87R mutant showed a 50-fold increase in the LD_50_ in a mouse peritonitis model, while the K42N and K42T mutants showed a 1,000-fold increase. The reduced virulence of these mutants should be partially caused by metabolic fitness, which was indicated by their slower growth than the parental strain when cultured in LB broth and decomplemented 75% NHS. The slower growth rate may cause them to be more easily eliminated by the immune system *in vivo*.

In the serum bactericidal assay, the K42N and K42T mutants were found to become susceptible to serum, while the alternative pathway should be the crucial role in complement activation. Whether this result is due to different levels of protein expression caused by the *rpsL* mutations requires further study, while this difference has been found between the K42T mutant and its parental strain in *E. carotovora* using a quantitative proteomic analysis [26]. Becoming serum-susceptible should explain the lowest virulence found for the K42N and K42T mutants compared with the parental strain, as well as the K42R and K87R mutants, which all showed serum resistance.

Based on the *in vitro* and animal experiments, the K42R mutant only showed a small fitness cost in a highly sensitive *in vitro* growth competition experiment. This mutant is also found at the highest frequency in streptomycin-resistant clinical isolates of *Mycobacterium tuberculosis*, while the K87R mutant was also prevalent [9], [45]. Because the properties of Arginine are very similar to that of Lysine, such as they both are positively-charged amino acids, this substitution could cause the subtle effect on protein structure and function [46].

Compared with the parental strain, all four streptomycin-resistant mutants exhibited reduced fitness, and three of them showed decreased virulence, while the ten streptomycin-dependent mutants needed streptomycin to maintain their growth. Only the K42R mutant showed similar pathogenicity to its parental strain as previously described in other *Enterobacteriaceae* [26], [27], [47]. This similar pathogenicity can also been found in a highly virulent *K. pneumoniae* strain; its K42R mutant has an LD_50_<10 CFU in a mouse peritonitis model as its parental strain and was used to construct unmarked in-frame gene deletions in our previous study [15]. In conclusion, the streptomycin resistance caused by *rpsL* mutations usually made the bacteria less competitive than its wild-type strain in the absence of streptomycin. The K42R mutant should be one of the best choices when using *rpsL* as a counter-selection marker.

## Supporting Information

Figure S1 **Creation of revertant strains for** ***rpsL*** **mutants.**
(TIF)

Table S1 **Oligonucleotide primers used in this study.**
(DOCX)
