# Supplementary figures and images for: A Suitable Streptomycin-Resistant Mutant for Constructing Unmarked In-Frame Gene Deletions Using rpsL as a Counter-Selection Marker
Source: PLoS One. 2014 Sep 30;9(9):e109258. doi: 10.1371/journal.pone.0109258 (PMC4182516; doi:10.1371/journal.pone.0109258)

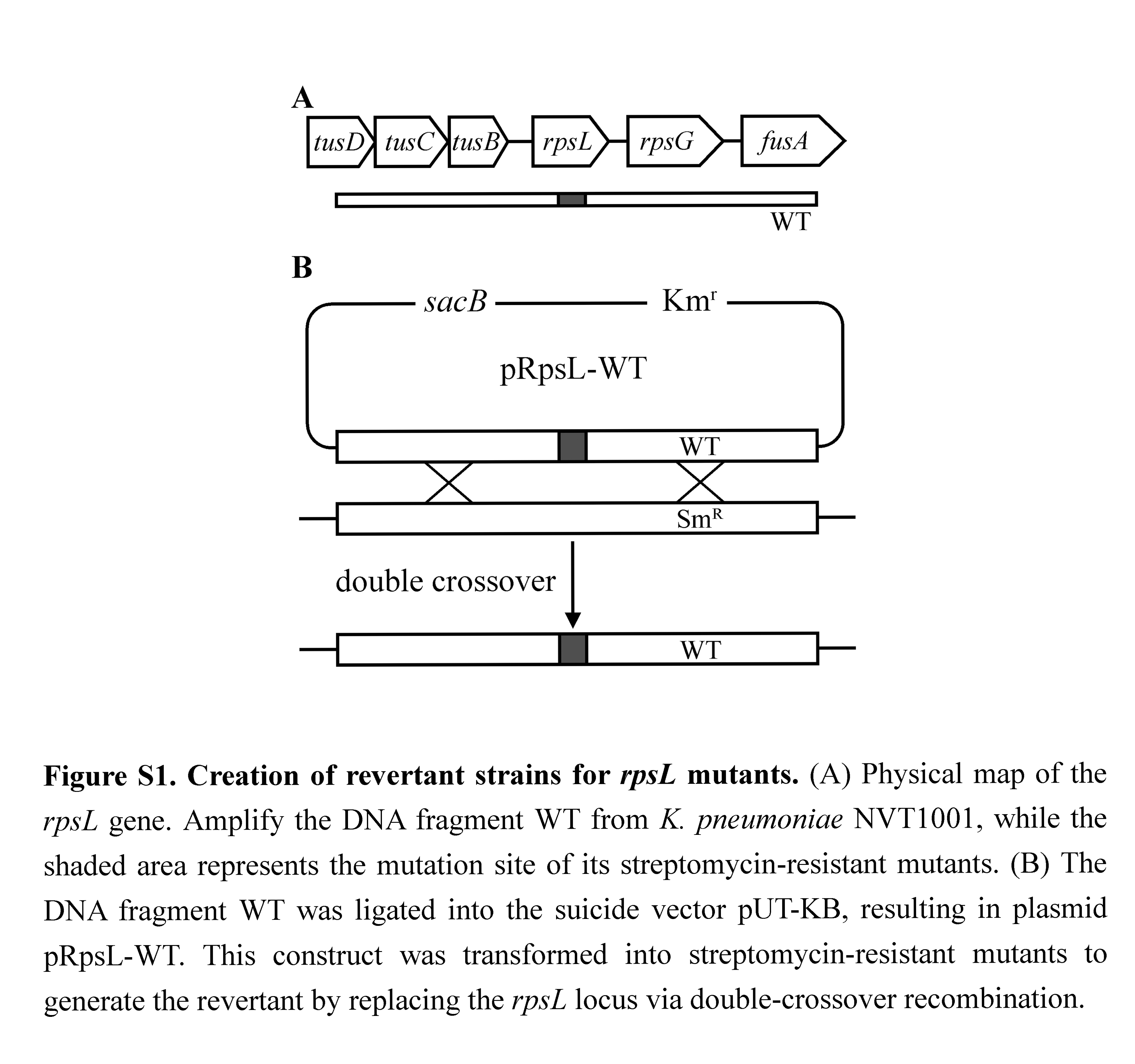

Supplement: Figure S1 — Creation of revertant strains for rpsL mutants. (TIF) [file pone.0109258.s001.tif]
