# Supplementary material for: A Suitable Streptomycin-Resistant Mutant for Constructing Unmarked In-Frame Gene Deletions Using rpsL as a Counter-Selection Marker
Source: PLoS One. 2014 Sep 30;9(9):e109258. doi: 10.1371/journal.pone.0109258 (PMC4182516; doi:10.1371/journal.pone.0109258)
Supplement: Table S1 — Oligonucleotide primers used in this study. (DOCX) [file pone.0109258.s002.docx]

**Table S1.** Oligonucleotide primers used in this study.

| Gene amplified | Primer | Sequence (5′-3′)*^a^* | Expected amplicon | Source or Reference |
| --- | --- | --- | --- | --- |
| *aadA* | aadA.for | GAGAACATAGCGTTGCCTTGG | 198 bp | [1] |
|  | aadA.rev | TCGGCGCGATTTTGCCGGTTAC |  | [1] |
| *strA* | strA.for | cttggtgataacggcaattc | 548 bp | [2] |
|  | strA.rev | CCAATCGCAGATAGAAGGC |  | [2] |
| *strB* | strB.for | ATCGTCAAGGGATTGAAACC | 509 bp | [2] |
|  | strB.rev | GGATCGTAGAACATATTGGC |  | [2] |
| *rpsL* and its flanking region (for detecting and sequencing) | rpsL.for | tccatacgctcagtgtttc | 911 bp | This study |
|  | rpsL.rev | TTACGCTGACCAATGACG |  | This study |
| *rpsL* and its flanking region (for constructing revertant) | ApaL.for | ACCGTGCACGGCAATCCCTCCACGAT (ApaLI) | 2401 bp | This study |
|  | ApaL.rev | ACCGTGCACCAGACTGCGGCTGAACAC (ApaLI) |  | This study |

*^a^* The restriction sites designed in the oligonucleotide are underlined, with the corresponding endonuclease indicated in parentheses.

**References**

1. Sunde M, Norström M (2005) The genetic background for streptomycin resistance in *Escherichia coli* influences the distribution of MICs. J Antimicrob Chemother 56: 87-90.

2. Gebreyes WA, Altier C (2002) Molecular characterization of multidrug-resistant *Salmonella enterica* subsp. *enterica* Serovar Typhimurium isolates from swine. J Clin Microbiol 40: 2813-2822.
